# Lipid-adiposity indices outperform traditional and derived anthropometric and biochemical indices for discriminating individuals with metabolic syndrome

**DOI:** 10.3389/fendo.2026.1901552

**Published:** 2026-07-30

**Authors:** Ahmad A. Obeidat, Mousa N. Ahmad, Salam M. Habib, Dalia Abu Al-Haijaa, Ohaad F. Awlya, Walaa E. Alhassani, Najlaa H. Almohmadi, Areej A. Almuraee, Firas S. Azzeh

**Affiliations:** 1 Human Nutrition and Dietetics, Department of Nutrition and Food Technology, The University of Jordan, Amman, Jordan; 2 Department of Diet Therapy Technology and Dietetics, Faculty of Allied Medical Sciences, Zarqa University, Al-Zarqa, Jordan; 3 Clinical Nutrition Department, Faculty of Applied Medical Sciences, Umm Al-Qura University, Makkah, Saudi Arabia; 4 Department of Biology, Preparatory Year Program, Batterjee Medical College, Jeddah, Saudi Arabia

**Keywords:** adiposity, anthropometry, derived indices, metabolic syndrome, receiver operating characteristic curves, sex-specific analysis

## Abstract

**Background:**

The most common traditional anthropometric indices used for metabolic syndrome (MetS) screening may not capture the metabolic risks associated with central adiposity, lipid dysregulation, and sex-specific metabolic risk. The purpose of this study was to compare traditional and derived anthropometric and biochemical parameters in the diagnosis of MetS among Jordanian adults.

**Methods:**

In this cross-sectional study, 930 adults (458 men and 472 women), aged 20–70 years, were assessed using harmonized IDF and AHA/NHLBI criteria. Among the traditional anthropometric indices, body mass index (BMI), waist circumference (WC), waist-to-height ratio (WHtR), and waist-to-hip ratio (WHR) were included. Several derived indices were computed: lipid accumulation product (LAP), visceral adiposity index (VAI), body roundness index (BRI), abdominal volume index (AVI), atherogenic index of plasma (AIP), cardiac risk index (CRI), and related lipid ratios. The diagnostic accuracy was verified using receiver operating characteristic (ROC) curve analysis, sensitivity, specificity, and the Youden index in sex-stratified analyses.

**Results:**

Among traditional indices, the results for WC and WHtR were good. The derived indices LAP and VAI were most accurate in both sexes, while the lipid ratios alone failed to provide good discrimination. BRI and AVI also showed good performance in derived anthropometric indices. Significant sex-specific differences were found in index distributions, optimal cutoff values, and their associations with MetS components.

**Conclusion:**

Derived lipid-adiposity indices are more discriminatory towards MetS than traditional single anthropometric indices, especially LAP and VAI. WC and WHtR are still good first-line screening tools while biochemical measurements may be used in the stratification of risk with LAP and VAI. The results of this study suggest the need for a multi-dimensional, sex-specific approach to screening for metabolic syndrome. The proposed cut-off values are based on a Jordanian population. Still, the approach of combining anthropometric, biochemical, and imaging-based surrogate markers for metabolic assessment may be transferable to other populations after external validation.

## 1 Introduction

Metabolic syndrome (MetS) represents a group of interrelated cardiometabolic abnormalities that raises the risk of cardiovascular disease (CVD) and type 2 diabetes mellitus (T2DM) (1). It is widely prevalent around the globe, and within regions, the burden of MetS is high amongst the Jordanian adult population (2). The early identification of individuals who are at risk for MetS is still crucial for prevention and timely action, as it can lead to morbidity, mortality, and increased healthcare costs (3).

The traditional anthropometric indices have been widely applied in clinical and epidemiological practice due to their simplicity, low cost, and non-invasive nature (4). These indices are body mass index (BMI), waist circumference (WC), waist to hip ratio (WHR), and waist to height ratio (WHtR). BMI is an indicator of overall body fatness, while WC, WHR, and WHtR indicate information about central fat distribution.

These single-measure indices, however, do not accurately capture the metabolic complexity of MetS, especially as body composition and recommended cut-off values differ between gender, age, and ethnicity. However, in Middle Eastern populations, universal thresholds might not be sufficient to establish cardiometabolic risk (5).

Previous studies in Jordan have shed light on anthropometric discriminators of MetS. Obeidat et al. (6) evaluated BMI, WC, WHR, and WHtR in Jordanian adults and reported that central obesity indices were more informative than BMI alone. Another study also evaluated anthropometric parameters and emphasized the importance of BRI and WHtR in the discrimination of MetS in the Jordanian population (5). These studies have laid the groundwork for population-specific screening, but most of them have concentrated on anthropometric measurements and have not examined in detail biochemical-integrated indices of adiposity and lipid metabolism.

The biological and clinical significance of sex-specific assessment is important because there are differences in fat distribution, hormonal regulation, lipid metabolism, and expression of cardiometabolic risk between women and men. Typical gender differences include accumulation of more visceral fat in men and more subcutaneous fat or gluteofemoral fat in women, and there may be differences in HDL-C and triglyceride patterns (7, 8). Therefore, cut-off values for sex stratified analysis might differ and enhance clinical interpretation of indices of screening for MetS.

To enhance risk discrimination using anthropometric and biochemical data, derived indices were developed, such as lipid accumulation product (LAP), visceral adiposity index (VAI), body roundness index (BRI), abdominal volume index (AVI), atherogenic index of plasma (AIP), cardiac risk index (CRI), and atherogenic coefficient (AC) (9, 10). These indices may better correlate with visceral adiposity, fat deposition, and atherogenic risk than are the traditional indices alone. Thus, the current study aimed to compare traditional and derived anthropometric and biochemical indices for the discrimination of MetS in Jordanian adults and to identify their optimal cut-off points and their correlations with the components of MetS using ROC curve analysis, and more specifically, the diagnostic performance across sexes.

## 2 Materials and methods

### 2.1 Study design and subjects

A total of 930 adults (458 males and 472 females) aged 20–70 years were selected in this cross-sectional study, from various areas of Jordan. Participants were enrolled using sex-stratified sampling to allow direct comparison of anthropometric and biochemical indices between men and women (11). MetS was diagnosed according to the harmonized IDF and AHA/NHLBI criteria (12). Exclusion criteria included pregnancy, lactation, severe acute illness, dementia, physical disability affecting anthropometric measurements, or incomplete anthropometric or biochemical data. A sample of 930 participants was considered adequate for sex-stratified ROC analyses and for comparing multiple screening indices because both male and female subgroups included more than 450 participants.

A standardized questionnaire was used to assess physical activity (PA) and was completed by trained interviewers. Participants reported how often and how long they engaged in physical activity during their work, transportation, home, and leisure time in the last seven days. Based on their answers, they were divided into three groups: Low physical activity (1), moderate (2), and high physical activity (3). The PA score was considered an ordinal variable for the purposes of description and comparison. This categorization is in line with the physical activity classification used in previous studies with the Jordanian population. Records with missing values for variables required to calculate a given index were excluded from that specific analysis; no imputation was applied.

### 2.2 Anthropometric measurements for obesity phenotyping and metabolic risk assessment

#### 2.2.1 Traditional anthropometric indices

Trained researchers measured all anthropometric variables using standardized procedures (13). Body weight (Wt) and height (Ht) were recorded with participants standing barefoot and wearing light clothing, using a calibrated digital scale and stadiometer (HGM-15, Shengyuan, China). Waist circumference (WC) was measured midway between the lowest rib margin and the iliac crest, and hip circumference (HC) was measured at the widest point of the gluteal region. Measurements were taken with participants standing upright and breathing normally. Traditional indices were calculated as follows: BMI = weight (kg)/height (m)^2; WHR = WC/HC; and WHtR = WC/height.

#### 2.2.2 Derived anthropometric indices

Derived anthropometric indices included body roundness index (BRI), a body shape index (ABSI), log-transformed ABSI (LBSI), log-transformed ABSI z-score (LBSIz), body adiposity index (BAI), conicity index (CI), abdominal volume index (AVI), ponderal index (PI), and waist-to-hip-to-height ratio (WHHR) (14–16). BRI and ABSI were calculated using established geometric equations (15), whereas BAI, CI, AVI, PI, and WHHR were calculated using standard formulae (16). The principal formulae were: BRI = 364.2 - 365.5 x sqrt[1 - (WC/(2pi))^2/(0.5 x Ht)^2]; ABSI = WC/[BMI^(2/3) x Ht^(1/2)]; LBSI = ln(ABSI); LBSIz = standardized z-score of LBSI; BAI = [HC/Ht^(1.5)] - 18; CI = WC/[0.109 x sqrt(Wt/Ht)]; AVI = [2 x WC^2 + 0.7 x (WC - HC)^2]/1000; PI = Wt/Ht^3; and WHHR = WHR/Ht. WC, HC, and height were used in consistent units for each equation. Derived anthropometric indices like Body Roundness Index (BRI) and Abdominal Volume Index (AVI) have been used in clinical interpretation for estimating body roundness and abdominal volume. These indices have shown correlation with the percentage of visceral fat and cardiometabolic risk. While ABSI can be used as a body shape indicator independent of size, its utility for screening for metabolic syndrome can differ by population. The percentage of body fat determined by BAI is based on hip circumference and height, while the percentage of body fat determined by CI is based on abdominal fat accumulation in relation to body mass and height. PI and WHHR are additional surrogate markers for adiposity as related to height or hip size.

### 2.3 Biochemical measurements for obesity phenotyping and metabolic risk assessment

Systolic blood pressure (SBP) and diastolic blood pressure (DBP) were measured from the right arm after participants rested quietly for at least 15 minutes using a standard mercury sphygmomanometer. Hypertension was defined as SBP >=130 mmHg and/or DBP >=85 mmHg, or current antihypertensive treatment (11, 12). Fasting blood samples were collected after an overnight fast of 10–12 hours. Fasting blood sugar (FBS), triglycerides (TG) (TRIG, Siemens, UK), total cholesterol (TC) (SPOTCHEM, ARKRAY, Japan), high-density lipoprotein cholesterol (HDL-C) (HDL, Kyowa Medex, Japan), and low-density lipoprotein cholesterol (LDL-C) (LDL kit, Minaris Medical, Japan) were measured using standard biochemical kits in an accredited clinical laboratory (12). Traditional biochemical variables included SBP, DBP, FBS, HDL-C, and TG. Derived biochemical indices included LAP, VAI, AIP, CRI, AC, HDL/LDL ratio, and TC/TG ratio (9, 10, 17). The principal formulae were: LAP = (WC - 65) x TG for men and (WC - 58) x TG for women; VAI = [WC/(39.68 + 1.88 x BMI)] x (TG/1.03) x (1.31/HDL-C) for men and [WC/(36.58 + 1.89 x BMI)] x (TG/0.81) x (1.52/HDL-C) for women; AIP = log10(TG/HDL-C); CRI = TC/HDL-C; AC = (TC - HDL-C)/HDL-C; HDL/LDL ratio = HDL-C/LDL-C; and TC/TG ratio = TC/TG. Lipid values were converted to appropriate molar units where required by the original equation. Derived biochemical indices: LAP combines WC and triglycerides as a better surrogate marker for central adiposity, lipid overload, and is a powerful surrogate for visceral fat dysfunction. VAI is a mathematical model which includes both adiposity (WC, BMI) and lipid parameters (TG, HDL-C) that are sex specific and estimate the function of VAT and insulin sensitivity. AIP is a description of the atherogenic nature of the plasma lipoprotein profile, and CRI and AC are expressions of the cholesterol-related atherogenic risk. The HDL/LDL and TC/TG ratios are individual lipid risk estimates that do not include adiposity surrogate markers. See **Supplementary Table 1** for a summary of all index definitions, formulae and physiological interpretations.

### 2.4 Metabolic syndrome definition

MetS was defined according to the harmonized IDF and AHA/NHLBI joint criteria (12), requiring the presence of three or more of the following risk factors (1): abdominal obesity based on waist circumference cut-offs of >=94 cm for men and >=80 cm for women, as adopted for Middle Eastern populations in the absence of country-specific thresholds (2); hypertriglyceridemia (>=1.7 mmol/L [>=150 mg/dL] or lipid-lowering treatment) (3); low HDL-C (<1.0 mmol/L [<40 mg/dL] in men or <1.3 mmol/L [<50 mg/dL] in women, or treatment) (4); elevated blood pressure (>=130/85 mmHg or antihypertensive treatment); and (5) elevated fasting glucose (>=5.6 mmol/L [>=100 mg/dL] or diagnosed T2DM).

### 2.5 Ethical Statement

All participants provided written informed consent after receiving information about the study objectives and procedures. Participants were informed that participation was voluntary and that they could withdraw at any time. The study was conducted in accordance with the Declaration of Helsinki and was approved by the Review Board of King Abdullah University Hospital, Endocrinology Clinics, Jordan (KAUH 1/145/2021; approval date: 2/12/2021).

### 2.6 Statistical analysis

Data were analyzed using STATA 15 (StataCorp, TX, USA). Continuous variables were presented as mean +/- standard error of the mean (SEM). Sex-based differences in baseline characteristics were assessed using independent-samples t tests. Analyses were performed separately for men and women because of known sex differences in body composition, fat distribution, and metabolic risk. The associations between indices and components of MetS were examined using partial correlation analysis, adjusting for age (17, 18). Given the cross-sectional design of this study, the evaluated indices were assessed for their ability to discriminate between individuals with and without metabolic syndrome (MetS), rather than to predict its future development. The ability of the discriminative performance was assessed by receiver operating characteristic (ROC) curve analysis, area under the curve (AUC), sensitivity, specificity, and Youden index (18, 19). Several indices were assessed, and the findings were interpreted by considering the effect size, biological plausibility, and consistency of results across sexes, and not just on the basis of a single p-value. A p-value < 0.05 was considered statistically significant. Considering the final statistical revision, binary logistic regression is suggested as an extra data-dependent analysis to assess the independent association of the most powerful indices with MetS status at the individual level.

## 3 Results

### 3.1 Characteristics of study participants: sex-based comparisons

**Table 1** summarizes the anthropometric and biochemical characteristics of 930 participants (458 men and 472 women). Men had significantly greater height, weight, WC, WHR, physical activity score, and TG levels, whereas women had significantly greater HC, BMI, WHtR, and HDL-C levels. Age, SBP, DBP, FBS, TC, and LDL-C showed no statistically significant sex differences based on the available summary statistics. These findings support the need for sex-stratified analyses because men and women differed in body size, fat distribution, and selected lipid parameters.

**Table 1:** Baseline anthropometric and biochemical characteristics of study participants by sex.

Indices	Mean ± standard error of mean (SEM)			Sex comparison	Sex comparison
	Male (458)	Female (472)	Total (930)	t-value	P-value
Age	44.51 ± 0.58	45.48 ± 0.60	45.00 ± 0.42	-1.16	0.245
PA	1.59 ± 0.02	1.49 ± 0.02	1.54 ± 0.18	3.54	<0.001
Ht (cm)	172.92 ± 0.33	159.28 ± 0.28	166.00 ± 0.31	31.52	<0.001
Wt (kg)	90.97 ± 0.93	80.64 ± 0.92	85.73 ± 0.68	7.90	<0.001
WC (cm)	104.68 ± 0.71	102.64 ± 2.37	103.64 ± 1.25	0.82	0.410
HC (cm)	109.24 ± 0.51	114.60 ± 0.67	111.96 ± 0.43	-6.37	<0.001
SBP	131.93 ± 1.24	132.47 ± 1.08	132.20 ± 0.82	-0.33	0.743
DBP	80.82 ± 0.52	81.41 ± 0.49	81.12 ± 0.36	-0.83	0.409
FBS (mg/dl)	119.68 ± 2.54	117.11 ± 2.66	118.38 ± 1.84	0.70	0.485
TC (mg.dl)	189.08 ± 1.88	194.16 ± 1.86	191.66 ± 1.33	-1.92	0.055
HDL (mg/dl)	45.11 ± 0.62	50.52 ± 0.65	47.86 ± 0.46	-6.02	<0.001
LDL (md/dl)	111.11 ± 1.72	112.19 ± 1.94	111.66 ± 1.30	-0.42	0.677
TG (mg/dl)	167.61 ± 3.66	150.72 ± 3.86	159.04 ± 2.68	3.18	0.002
Ht (m)	1.73 ± 0.00	1.59 ± 0.00	1.66 ± 0.00	NA	NA
BMI (kg/m2)	30.37 ± 0.29	31.85 ± 0.36	31.12 ± 0.23	-3.20	0.001
WHtR	0.61 ± 0.00	0.65 ± 0.02	0.63 ± 0.01	-2.00	0.046
WHR	0.96 ± 0.00	0.89 ± 0.02	0.92 ± 0.01	3.50	<0.001

PA, physical activity score (1 = low/sedentary, 2 = moderate, 3 = high); Ht, height; Wt, weight; WC, waist circumference; HC, hip circumference; SBP, systolic blood pressure; DBP, diastolic blood pressure; FBS, fasting blood sugar; TC, total cholesterol; HDL-C, high-density lipoprotein cholesterol; LDL-C, low-density lipoprotein cholesterol; TG, triglycerides; BMI, body mass index; WHtR, waist-to-height ratio; WHR, waist-to-hip ratio. P-values compare males and females using independent-samples t tests based on mean ± SEM values.

### 3.2 Anthropometric indices

**Figure 1:**
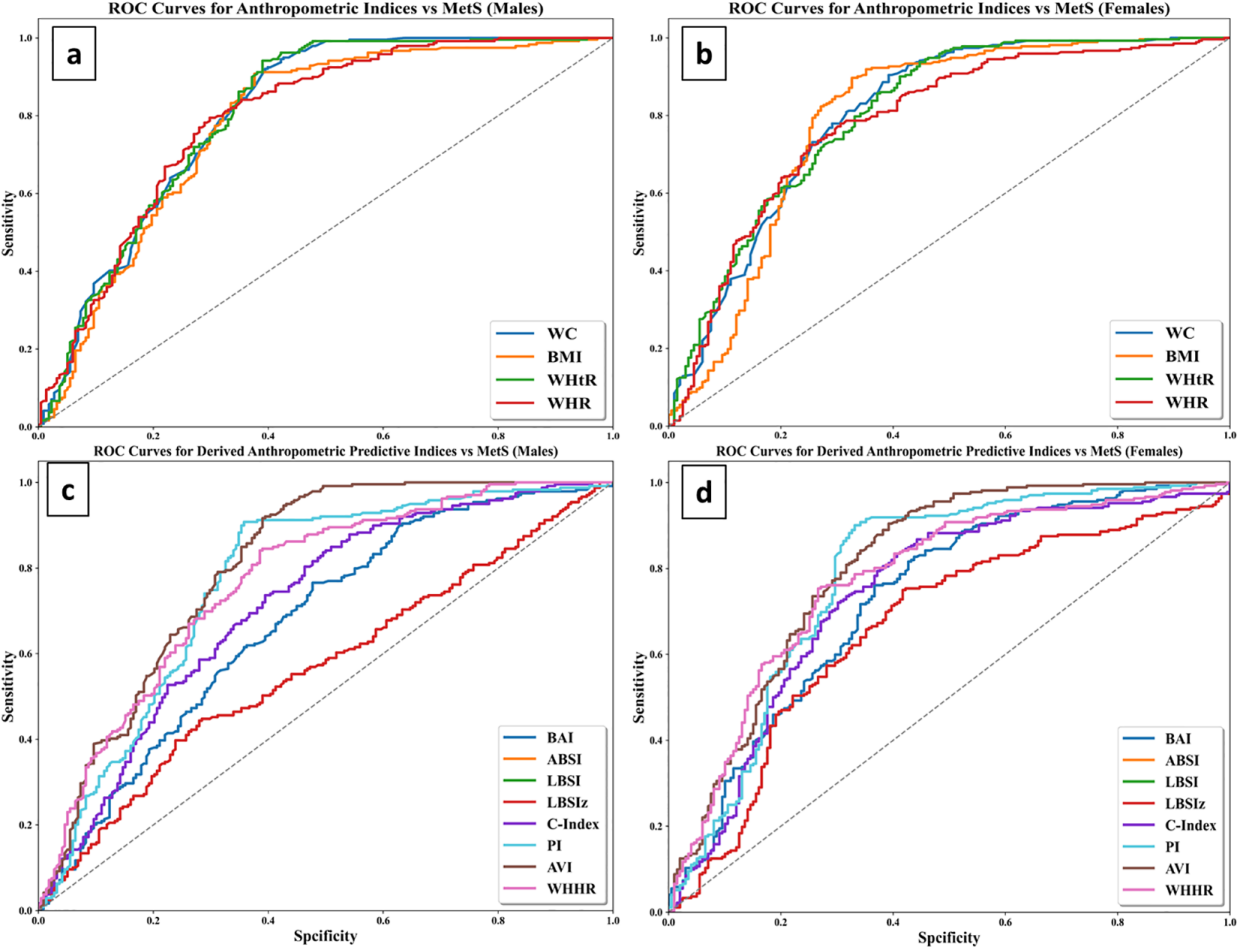
ROC curves for traditional and derived anthropometric indices: **(A)** traditional anthropometric indices vs. MetS in males; **(B)** traditional anthropometric indices vs. MetS in females; **(C)** derived anthropometric indices vs. MetS in males; and **(D)** derived anthropometric indices vs. MetS in females.

Among traditional anthropometric indices, WC and WHtR showed the strongest discrimination for MetS in both sexes (**Table 2**; **Figure 1**). WC had identical AUC values in men and women (AUC = 0.807), with high sensitivity in men and balanced sensitivity and specificity in women. WHtR also showed strong and similar discriminative performance in men and women. BMI performed moderately, whereas WHR showed comparatively lower discrimination among traditional indices. These results indicate that central adiposity indices provide better MetS discrimination than general adiposity alone.

**Table 2:** Traditional anthropometric indices for discriminating individuals with metabolic syndrome in males and females.

Parameter	Cutoff point		AUC		Youden index		P-value
	Male	Female	Male	Female	Male	Female	(M/F)
WC (cm)	97.3	97.55	0.807	0.807	0.521	0.499	<0.001
BMI (kg/m²)	29.8	30.65	0.780	0.792	0.529	0.545	<0.001
WHtR	0.565	0.615	0.806	0.808	0.553	0.469	<0.001
WHR	0.955	0.855	0.794	0.786	0.467	0.474	<0.001

WC, waist circumference; BMI, body mass index; WHtR, waist-to-height ratio; WHR, waist-to-hip ratio; AUC, area under the ROC curve.

Among derived anthropometric indices, AVI and BRI were the strongest performers, with AUC values greater than 0.80 in both sexes (**Table 3**; **Figure 1**). BRI showed particularly high sensitivity in men, whereas AVI provided balanced performance across sexes. PI, CI, BAI, and WHHR showed moderate discrimination, whereas ABSI, LBSI, and LBSIz had limited discriminative value, especially in men. These findings suggest that volume- and roundness-based indices may be better surrogate markers for central adiposity than shape indices alone.

**Table 3:** Derived anthropometric indices for discriminating individuals with metabolic syndrome in males and females.

Parameter	Cutoff point		AUC		Sensitivity		Specificity			Youden index		
	Male	Female	Male	Female	Male	Female	Male	Female		Male	Female	
BRI	4.657	5.247	0.806	0.804	0.941	0.856	0.612		0.632	0.553	0.488	
ABSI	0.083	0.076	0.569	0.670	0.448	0.753	0.712		0.582	0.16	0.335	
LBSI	-1.082	-1.118	0.569	0.670	0.448	0.753	0.712		0.582	0.16	0.335	
LBSIz	19.311	19.270	0.569	0.670	0.402	0.756	0.744		0.577	0.146	0.333	
BAI	28.600	36.542	0.673	0.731	0.762	0.768	0.521		0.617	0.283	0.385	
CI	1.298	1.254	0.715	0.743	0.736	0.756	0.607		0.652	0.343	0.408	
AVI	19.525	18.730	0.806	0.806	0.921	0.845	0.607		0.662	0.528	0.507	
PI	16.480	18.045	0.770	0.782	0.908	0.904	0.644		0.657	0.552	0.561	
WHHR	0.536	0.541	0.770	0.778	0.845	0.764	0.612		0.716	0.457	0.48	

BRI, body roundness index; ABSI, a body shape index; LBSI, log-transformed body shape index; LBSIz, log-transformed body shape index z-score; BAI, body adiposity index; CI, conicity index; AVI, abdominal volume index; PI, ponderal index; WHHR, waist-to-hip-to-height ratio. Significance level, p < 0.001 unless otherwise stated.

The age-adjusted correlation analysis showed sex-specific associations between anthropometric indices and MetS components (**Table 4**). In men, WC, BMI, WHtR, and WHR were positively correlated with blood pressure, FBS, TG, and TC and negatively correlated with HDL-C. In women, BMI showed the strongest correlations with SBP and DBP, whereas waist-based indices showed weaker correlations with lipid variables. These results suggest that waist-based indices are more closely linked to visceral and lipid-related risk in men, whereas BMI may capture broader adiposity-related risk in women.

**Table 4:** Age-adjusted partial correlation coefficients between traditional anthropometric indices and metabolic syndrome risk factors.

Indices		Correlation coefficient (r)			
		WC	BMI	WHtR	WHR
SBP	Male	0.261***	0.282***	0.256***	0.298***
	Female	0.171***	0.467***	0.172***	0.103*
	Total	0.163***	0.369***	0.155***	0.112***
DBP	Male	0.313***	0.283***	0.309***	0.313***
	Female	0.202***	0.406***	0.200***	0.142**
	Total	0.197***	0.347***	0.189***	0.140***
FBS	Male	0.267***	0.311***	0.281***	0.286***
	Female	0.180***	0.270***	0.182***	0.144**
	Total	0.181***	0.286***	0.175***	0.147***
TG	Male	0.176***	0.222***	0.171***	0.160**
	Female	0.063	0.117*	0.063	0.045
	Total	0.090**	0.159***	0.073*	0.072*
TC	Male	0.177***	0.155**	0.168***	0.102*
	Female	0.060	-0.012	0.060	0.049
	Total	0.081*	0.075*	0.082*	0.045*
HDL-C	Male	-0.394***	-0.381***	-0.367***	-0.372***
	Female	-0.075	-0.092*	-0.065	-0.058
	Total	-0.124***	-0.180***	-0.082*	-0.110**

*p < 0.05; **p < 0.01; ***p < 0.001. WC, waist circumference; BMI, body mass index; WHtR, waist-to-height ratio; WHR, waist-to-hip ratio; SBP, systolic blood pressure; DBP, diastolic blood pressure; FBS, fasting blood sugar; HDL-C, high-density lipoprotein cholesterol; TG, triglycerides; TC, total cholesterol.

### 3.3 Biochemical indices

**Figure 2:**
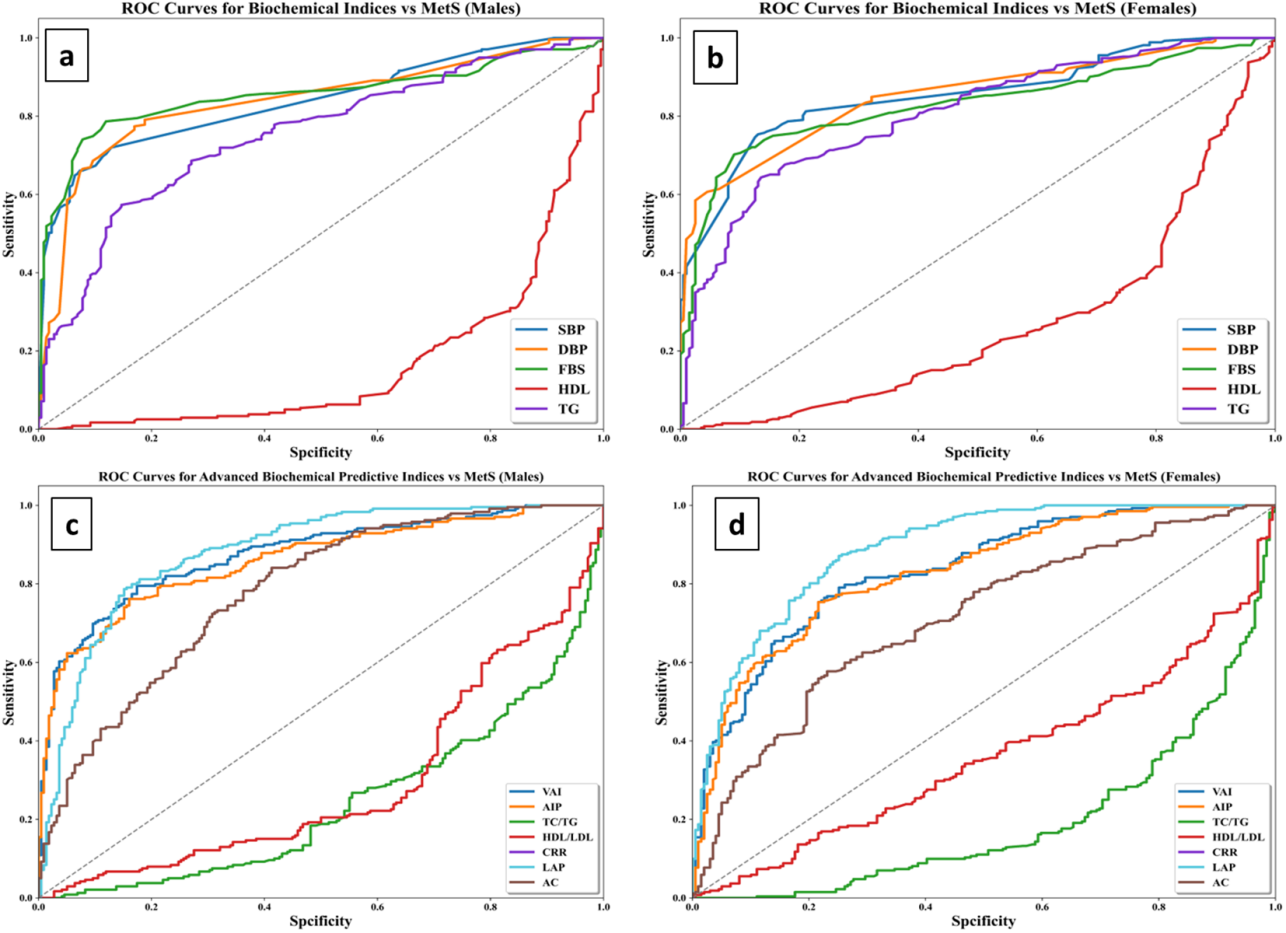
ROC curves for traditional and derived biochemical indices: **(A)** traditional biochemical variables vs. MetS in males; **(B)** traditional biochemical variables vs. MetS in females; **(C)** derived biochemical indices vs. MetS in males; and **(D)** derived biochemical indices vs. MetS in females.

**Table 1** shows that men had higher TG levels than women, whereas women had higher HDL-C levels. The ROC analysis of traditional biochemical variables showed that FBS, SBP, DBP, and TG had clinically meaningful discrimination for MetS, whereas HDL-C alone showed weak discrimination (**Table 5**; **Figure 2**).

**Table 5:** Traditional biochemical variables for discriminating individuals with metabolic syndrome in males and females.

Parameter	Cut-off		AUC		Youden index		P-value
	Male	Female	Male	Female	Male	Female	(M/F)
SBP (mmHg)	121.0	120.5	0.839	0.851	0.592	0.598	<0.001
DBP (mmHg)	77.5	75.5	0.838	0.852	0.591	0.529	<0.001
FBS (mg/dL)	99.5	94.5	0.854	0.827	0.668	0.585	<0.001
HDL (mg/dL)	63.5	66.5	0.167	0.269	-0.149	-0.150	<0.001
TG (mg/dL)	157.5	140.5	0.753	0.807	0.417	0.505	<0.001

SBP, systolic blood pressure; DBP, diastolic blood pressure; FBS, fasting blood sugar; HDL-C, high-density lipoprotein cholesterol; TG, triglycerides; AUC, area under the ROC curve.

Among derived biochemical indices, LAP and VAI demonstrated the highest diagnostic performance for MetS in both sexes (**Table 6**; **Figure 2**). In men, LAP had the highest discriminative accuracy, followed closely by VAI and AIP. For women, LAP had the best results, followed by VAI and AIP. Conversely, HDL/LDL and TC/TG ratios showed poor discrimination in both sexes. The results indicate that LAs are more informative than individual lipid ratios, as they include lipid dysregulation and central adiposity.

**Table 6:** Derived biochemical indices for discriminating individuals with metabolic syndrome in males and females.

Parameter	Cutoff point		AUC		Sensitivity		Specificity			Youden index		
	Male	Female	Male	Female	Male	Female	Male	Female		Male	Female	
LAP	319.73	267.81	0.881	0.889	0.812	0.849	0.822		0.776	0.634	0.625	
VAI	4.717	4.557	0.875	0.836	0.795	0.797	0.826		0.751	0.621	0.548	
AIP	0.528	0.408	0.860	0.829	0.795	0.756	0.785		0.781	0.58	0.537	
CRI	3.862	3.640	0.780	0.708	0.782	0.679	0.639		0.612	0.421	0.291	
AC	2.685	2.640	0.780	0.708	0.841	0.679	0.589		0.612	0.43	0.291	
HDL/LDL ratio	0.627	0.616	0.291	0.368	0.117	0.232	0.721		0.677	-0.162	-0.091	
TC/TG ratio	1.546	2.080	0.243	0.190	0.117	0.096	0.557		0.597	-0.326	-0.307	

LAP, lipid accumulation product; VAI, visceral adiposity index; AIP, atherogenic index of plasma; CRI, cardiac risk index; AC, atherogenic coefficient; HDL/LDL ratio, high-density lipoprotein cholesterol/low-density lipoprotein cholesterol ratio; TC/TG ratio, total cholesterol/triglyceride ratio. Significance level, p < 0.001 unless otherwise stated.

Results from the correlations further show sex specific metabolic patterns (**Table 4**). In men, adiposity indices had greater negative associations with HDL-C and positive associations with TG and blood pressure, indicating a greater association of visceral adiposity with metabolic dysfunction. Correlation between the waist-based measurement and lipid variables was weaker in women, while BMI had stronger relationships with blood pressure. These patterns provide a rationale for the biological basis for sex-stratified interpretation of MetS screening indices.

## 4 Discussion

The superiority of WC and WHtR in discriminating individuals with metabolic syndrome compared to BMI can be attributed to their anatomic and physiologic relationship to the visceral adipose tissue (VAT). Visceral adipose tissue (VAT) differs from subcutaneous adipose tissue, which has relatively low metabolic activity and a direct drainage pathway to the portal circulation via the splanchnic bed. This anatomical arrangement is associated with high levels of free fatty acids entering the liver and contributing to insulin resistance, increased very-low-density lipoproteins (VLDL) production and dyslipidemia (raised triglycerides and lower HDL cholesterol). Furthermore, VAT has been shown to be a major source of pro-inflammatory adipokines (tumor necrosis factor-α, interleukin-6, resistin and plasminogen activator inhibitor-1) and to have reduced secretion of the protective adipokine, adiponectin. Such secretory patterns help to maintain low-grade inflammation, endothelial dysfunction and glucose dysregulation, which are hallmark features of metabolic syndrome. Because BMI is an indicator of total body mass and does not distinguish between different types of body fat, lean mass or fat distribution, it fails to separate out metabolically harmful visceral fat from relatively less harmful subcutaneous fat. Waist circumference (WC) is a direct indicator of the amount of abdominal fat, while waist-to-height ratio (WHtR) normalizes the measure for body height. This normalization increases the ability to compare between the data of contrasting sexes, ages, and ethnic groups. Therefore, WC and WHtR are more physiologically meaningful surrogate markers for ectopic fat accumulation and adipose tissue dysfunction, at the core of metabolic syndrome pathophysiology. LAP and the VAI are more powerful lipid-adiposity composite indices, further underscoring the importance of adding central adiposity to lipid dysregulation. These indices show the complex pathophysiological interactions between the increase of visceral fat and deposition of ectopic lipids, which are associated with the development of Metabolic Syndrome (MetS). They provide a more complex metabolic risk assessment than adiposity-based.

The present study aimed to assess the discriminative ability of traditional and derived anthropometric and biochemical indices for discriminating individuals with MetS among Jordanian adults and identified central adiposity and lipid-adiposity dysfunction as the most discriminating indices. WC and WHtR were superior to BMI and WHR in terms of traditional indices, thus further confirming the central role of abdominal adiposity in the pathophysiology of MetS. The results are similar to those of previous studies that have demonstrated that waist measurement better associated with the adiposity of the visceral stores and cardiometabolic risk compared to BMI alone (2, 7, 15).

The current results also corroborate previous studies conducted in Jordan. Al-Shami et al. (5) reported that WHtR and BRI were good anthropometric discriminators, while Obeidat et al. (6) found central obesity indices to be useful discriminators in Jordanian adults. The present study builds on this previous research by incorporating biochemical-based indices, specifically LAP, VAI, AIP, CRI, and AC, as well as assessing the ability of the lipid-integrated chemical indices to better identify risks than the anthropometric chemical indices alone. The improved performance of LAP and VAI indicates that TG and HDL-C add to the metabolic dysfunction independent of body size and body shape measures alone (20, 21).

Derived anthropometric indices, such as BRI and AVI, had good diagnostic performance, suggesting that indices related to body roundness and abdominal volume could be better suited as surrogate markers for visceral fat accumulation than simple ratios. The results of this study are in line with the previous reports showing that BRI can be helpful in providing information on body shape and central adiposity (2, 17, 22, 23). Conversely, ABSI and log-ABSI exhibited poor discrimination, especially among men, consistent with previous evidence of limited clinical value of using ABSI to screen for MetS in certain populations (2, 20, 24). Therefore, not every derived anthropometric index is clinically useful: some indices, such as indices related to the volume of the abdomen or lipid-adiposity interaction, seem more informative.

LAP and VAI are superior in performance and have biological plausibility. LAP is a combination of WC and TG, which is abdominal fat accumulation and lipid overload, respectively; VAI is an integration of WC together with BMI, TG, and HDL-C, which is associated with visceral adipose tissue dysfunction. Visceral adiposity has been shown to be tightly associated with insulin resistance, dysregulated adipokine production, low-grade inflammation, hypertriglyceridemia and decreased HDL-C, all of which are involved in MetS (20, 21). Therefore, the lipid adiposity indices may be better able to detect metabolic risk than any single indicator like BMI, HDL/LDL ratio or TC/TG ratio. In line with previous studies where lipid-integrated indices were better than the conventional anthropometric-based or lipid-based indices in detecting cardiometabolic risk (4, 21, 25, 26), the similar results were obtained in the current study.

### 4.1 Sex-specific differences

Sex-specific differences were evident in baseline characteristics, ROC-derived cut-offs, and correlation patterns. Men had higher WC, WHR, and TG levels, indicating a more centralized and atherogenic adiposity pattern, whereas women had higher BMI, HC, WHtR, and HDL-C. These differences support the reviewer-recommended subgroup analysis by sex and indicate that a single universal cut-off may not perform equally in both groups. The stronger associations between waist-based indices and TG, blood pressure, and HDL-C in men suggest that visceral fat accumulation is a major driver of metabolic risk (27, 28). In women, stronger BMI-blood pressure correlations may denote the influence of total adiposity, subcutaneous fat distribution, and hormonal regulation (17, 18). The findings are consistent with the recommendation to use sex-specific screening methods instead of using the same thresholds for men and women.

### 4.2 Clinical and public health implications

Clinically, WC and WHtR are still valid first line screening tests as these are inexpensive, quick and simple to apply in community and primary-care settings. If biochemical data is available, however, LAP and VAI may offer a more accurate risk stratification by integration of adiposity and lipid dysfunction. This method is especially useful when distinguishing those whose BMI may not reveal risk of metabolic disease. A sequential approach might be feasible in resource-poor areas – WC/WHtR as a screening test to calculate LAP or VAI for patients with borderline or high risk scores. This would provide a balance between feasibility, better diagnostic accuracy, and better sex-specific prevention.

### 4.3 Strengths and limitations

The strengths of this study include a relatively large sample size, sex-stratified analyses, evaluation of multiple traditional and derived indices, and use of ROC-based diagnostic performance markers. Lifestyle factors such as smoking, diet, physical activity, socioeconomic status, and possible seasonal variation in biochemical parameters were not fully incorporated into adjusted models. A major conceptual drawback of the present study is that all the indices assessed, including anthropometric indices and biochemical indices, are not direct measures of visceral adipose tissue (VAT). Waist circumference is used in the composite indices such as Lipid Accumulation Product (LAP) and Visceral Adiposity Index (VAI) for estimating visceral fat dysfunction. Despite this, these indices are not entirely representative of the amount of visceral adipose tissue (VAT), the presence of adipose tissue inflammation, the pattern of adipokine release, or the accumulation of ectopic fat in organs, including the liver, skeletal muscle, or pancreas. Thus, people with the same BMI or waist circumference can have very different metabolic risk profiles, and the concepts of “metabolically healthy obese” and “metabolically unhealthy normal weight” phenotypes have been established. Therefore, the improved diagnostic capabilities of LAP and VAI found in this study should be interpreted as an improvement in surrogate-based risk discrimination, not as an evaluation of visceral adiposity phenotypes. Logistic regression and external validation should be incorporated in future analyses when participant-level data are available.

### 4.4 Future directions: multimodal phenotyping and ultrasound-based assessment of visceral fat

Recent studies have shown that imaging-based evaluation of visceral adipose tissue can improve the definition of surrogate indices for the risk stratification of metabolic syndrome. In primary care and community settings, non-invasive ultrasound measurement of the thickness of the abdominal fat compartments (subcutaneous, preperitoneal, omental and perirenal fat thickness) presents a practical, radiation-free and economical approach to the direct phenotyping of visceral fat (29). Unlike anthropometric surrogates, ultrasound has the potential to distinguish between subcutaneous and visceral fat depots. This modality could also support the identification of those who do not have a BMI or waist circumference that is classified as overweight or obese, but who do have disproportionate amounts of visceral fat. Furthermore, shear-wave elastography is able to estimate liver steatosis and fibrosis simultaneously, which are commonly associated with visceral obesity and insulin resistance (29).

Multiparametric ultrasound is not just a tool for measuring visceral fat thickness but is also able to provide multiparametric metabolic phenotyping of abdominal fat compartments and target-organ involvement such as hepatic steatosis, liver fibrosis, renal changes, and other organ manifestations of metabolic syndrome. In a clinical setting, ultrasound should be used in combination with other laboratory markers and used as part of a panel of imaging tests. Imaging biomarkers and biochemical indices are complementary, yet may offer very different information, and their combination is a promising area for precision metabolic medicine and individualized cardiometabolic risk stratification.

Researchers have emphasized the fact that use of BMI alone can mask substantial metabolic risks in people who are of normal weight but who have high levels of visceral adiposity. This situation highlights the need for a multimodal assessment approach (30). So, future precision medicine interventions to treat metabolic syndrome should move beyond simple anthropometric categorizations. However, they should use an integrated phenotyping approach with methods of anthropometric screening (waist circumference, waist-to-height ratio), biochemical surrogate markers (lipid accumulation product, visceral adiposity index, atherogenic index of plasma), and imaging biomarkers (ultrasound-visceral adipose tissue, hepatic elastography). A multimodal assessment for metabolic syndrome should be used for future stratification. Comprehensive risk assessment models should combine anthropometric with biochemical surrogate markers, imaging biomarkers, and relevant clinical characteristics. The multimodal phenotyping has the potential to enhance personalized prediction, early detection, and preventative measures in various populations. This would be highly relevant to the Middle Eastern population where sex, age, and ethnicity have a significant impact on body composition and fat distribution, which are associated with metabolic risk. Future studies of Jordanian adults should incorporate measurement of ultrasound-visceral fat to validate and improve the cut-off values used in this study.

## 5 Conclusion

The present study shows that both lipid-adiposity indices (Lipid Accumulation Product-LAP and Visceral Adiposity Index-VAI) have greater discriminative power for metabolic syndrome than any single anthropometric index. The list of cut-off values provided is specific to this Jordanian population, but the conceptual approach and the emphasis on using a lipid-adiposity composite index rather than an isolated anthropometric measure, and using sex-specific multimodal screening, are generally applicable to other populations after appropriate external validation. WC and WHtR remain useful and cost-effective screening tools. Moreover, anthropometric indices derived from these measurements, like body roundness index (BRI) and abdominal volume index (AVI), are additional advantages. Findings must be interpreted on a sex-stratified basis because there are significant differences in the distribution of the indices, optimal index cut-off, and patterns of correlations between men and women. However, it is essential to understand that LAP, VAI, etc. are indirect indices of visceral adiposity, and cannot be used as a direct measurement. This means their results should be interpreted as a risk discrimination performance improvement and not as a direct assessment of metabolic phenotyping. Further research is needed to develop and validate comprehensive multimodal assessment models incorporating anthropometric measures, biochemical surrogate markers, imaging biomarkers (such as ultrasound assessment of abdominal fat compartments and hepatic elastography), and clinical characteristics in integrated risk assessment tools. The specific cut-off thresholds reported in this study need to be validated in an external population, but the overall approach of multimodal phenotyping, lipid-adiposity composite indices, imaging biomarkers, and sex-specific risk assessment is likely to be transferable to other populations and can be used to progress metabolic medicine toward precision medicine.

## Data Availability

The raw data supporting the conclusions of this article will be made available by the authors, without undue reservation.

## Supplementary material

Supplementary Table 1 Summary of index definitions, formulae, and physiological interpretations.
