# Supplementary material for: Lipid-adiposity indices outperform traditional and derived anthropometric and biochemical indices for discriminating individuals with metabolic syndrome
Source: Front Endocrinol (Lausanne). 2026 Jul 30;17:1901552. doi: 10.3389/fendo.2026.1901552 (PMC13467773; doi:10.3389/fendo.2026.1901552)
Supplement: Supplementary file 1 [file Table1.docx]

Supplementary Table S1. Summary of index definitions, formulae, and physiological interpretations.

| **Index** | **Full Name** | **Formula** | **Physiological Interpretation** |
| --- | --- | --- | --- |
| LAP | Lipid Accumulation Product | (WC – 65) × TG for men and (WC – 58) × TG for women | Indicator of lipid accumulation in visceral adipose tissue |
| VAI | Visceral Adiposity Index | [WC/(39.68 + 1.88 x BMI)] x (TG/1.03) x (1.31/HDL-C) for men and;  [WC/(36.58 + 1.89 x BMI)] x (TG/0.81) x (1.52/HDL-C) for women | Marker of visceral adipose tissue impairment and cardiometabolic risk |
| BRI | Body Roundness Index | 364.2 - 365.5 x sqrt[1 - (WC/(2pi))^2/(0.5 x Ht)^2] | Assesses body roundness and corresponds with visceral fat percentage |
| AVI | Abdominal Volume Index | [2 x WC^2 + 0.7 x (WC - HC)^2]/1000 | Calculates the abdominal volume index |
| ABSI | A Body Shape Index | WC/[BMI^(2/3) x Ht^(1/2)] | Represents bodily form irrespective of dimensions |
| BAI | Body Adiposity Index | [HC/Ht^(1.5)] - 18 | Calculates body fat percentage using hip circumference and height measurements |
| CI | Conicity Index | WC/[0.109 x sqrt(Wt/Ht)] | Measures abdominal fat accumulation in relation to body mass and height |
| AIP | Atherogenic Index of Plasma | log(TG/HDL-C) | Indicates the atherogenic propensity of the plasma lipoprotein profile |
| CRI | Castelli Risk Index | TC/HDL-C | Measures cholesterol-associated atherogenic risk |
| AC | Atherogenic Coefficient | (TC - HDL-C)/HDL-C | Alternative indication of atherogenic cholesterol risk |
